# The InBIO Barcoding Initiative Database: contribution to the knowledge on DNA barcodes of Iberian Plecoptera

**DOI:** 10.3897/BDJ.8.e55137

**Published:** 2020-07-07

**Authors:** Sonia Ferreira, José Manuel Tierno de Figueroa, Filipa MS Martins, Joana Verissimo, Lorenzo Quaglietta, José Manuel Grosso-Silva, Pedro B Lopes, Pedro Sousa, Joana Paupério, Nuno A Fonseca, Pedro Beja

**Affiliations:** 1 CIBIO-InBIO, Centro de Investigação em Biodiversidade e Recursos Genéticos, Universidade do Porto, Vairão, Portugal CIBIO-InBIO, Centro de Investigação em Biodiversidade e Recursos Genéticos, Universidade do Porto Vairão Portugal; 2 Universidad de Granada, Granada, Spain Universidad de Granada Granada Spain; 3 Departamento de Biologia, Faculdade de Ciências, Universidade do Porto, Porto, Portugal Departamento de Biologia, Faculdade de Ciências, Universidade do Porto Porto Portugal; 4 CIBIO-InBIO, Centro de Investigação em Biodiversidade e Recursos Genéticos, Instituto Superior de Agronomia, Lisboa, Portugal CIBIO-InBIO, Centro de Investigação em Biodiversidade e Recursos Genéticos, Instituto Superior de Agronomia Lisboa Portugal; 5 Museu de História Natural e da Ciência da Universidade do Porto, Porto, Portugal Museu de História Natural e da Ciência da Universidade do Porto Porto Portugal; 6 Rua do Torgal nº16, Trigais - Covilhã, 6215-295 Erada, Covilhã, Portugal Rua do Torgal nº16, Trigais - Covilhã, 6215-295 Erada Covilhã Portugal

**Keywords:** Plecoptera, occurrence records, species distributions, continental Portugal, continental Spain, DNA barcode, COI

## Abstract

**Background:**

The use of DNA barcoding allows unprecedented advances in biodiversity assessments and monitoring schemes of freshwater ecosystems; nevertheless, it requires the construction of comprehensive reference collections of DNA sequences that represent the existing biodiversity. Plecoptera are considered particularly good ecological indicators and one of the most endangered groups of insects, but very limited information on their DNA barcodes is available in public databases. Currently, less than 50% of the Iberian species are represented in BOLD.

**New information:**

The InBIO Barcoding Initiative Database: contribution to the knowledge on DNA barcodes of Iberian Plecoptera dataset contains records of 71 specimens of Plecoptera. All specimens have been morphologically identified to species level and belong to 29 species in total. This dataset contributes to the knowledge on the DNA barcodes and distribution of Plecoptera from the Iberian Peninsula and it is one of the IBI database public releases that makes available genetic and distribution data for a series of taxa.

The species represented in this dataset correspond to an addition to public databases of 17 species and 21 BINs. Fifty-eight specimens were collected in Portugal and 18 in Spain during the period of 2004 to 2018. All specimens are deposited in the IBI collection at CIBIO, Research Center in Biodiversity and Genetic Resources and their DNA barcodes are publicly available in the Barcode of Life Data System (BOLD) online database. The distribution dataset can be freely accessed through the Global Biodiversity Information Facility (GBIF).

## Introduction

In freshwater ecosystems, biodiversity assessments and monitoring schemes often require the identification of aquatic insect species (e.g. Pawlowski et al. 2018), an often challenging step, namely when only first instars are available in the sample or when studies are developed in regions poorly known from a faunistic perspective. In such cases, DNA barcoding provides a powerful tool to overcome these challenges by using a fragment of DNA to assign organisms to a species in a rapid and automated way (Hebert et al. 2003). Furthermore, environmental DNA (eDNA) is an emerging tool with great potential in conservation for monitoring past and present biodiversity, both in terrestrial and aquatic ecosystems (Thomsen and Willerslev 2015), especially when DNA barcode reference collections are used to link the obtained sequences to reliably identified organisms. The use of DNA barcoding requires the construction of comprehensive reference collections of DNA sequences that represent the existing biodiversity (Ferreira et al. 2018, Kress et al. 2005, Baird et al. 2011). In Europe, initiatives like the DNA barcoding projects, overseen by the Bavarian State Collection of Zoology in Munich (SNSB-ZSM—www.barcoding. zsm.de) through the “Barcoding Fauna Bavarica project” (BFB—www.faunabavarica.de—Haszprunar, 2009), launched in 2009 and by the “German Barcode of Life project” (GBOL—www.bolgermany.de), launched in 2012 (Geiger et al. 2016), has led to the public release of DNA barcode sequence data of over 300 species of Ephemeroptera, Plecoptera and Trichoptera (Morinière et al. 2017). As part of the Mediterranean Basin Biodiversity Hotspot, the Iberian Peninsula presents not only high numbers of species, as it also harbours species with limited distribution range, with many absent in central and northern Europe. The InBIO Barcoding Initiative (IBI) was established to overcome the striking scarcity of genetic data associated with the high biodiversity found in Portugal, focusing mainly on invertebrate taxa. Within the project, a special focus was afforded to aquatic insects, given their role as indicators in biodiversity assessments and monitoring schemes (e.g. Weisser and Siemann 2004, Weigand et al. 2019) and their relevance to food webs and ecosystem functioning. Furthermore, many insect species occurring in the Iberian Peninsula are not represented in public barcode databases (Ferreira et al. 2019, Ferreira et al. 2018, Weigand et al. 2019) and those that exist often show high evolutionary distances to the sequences obtained in this region which may indicate cryptic diversity (Corley et al. 2019b, Corley et al. 2019a, Ferreira et al. 2018). DNA barcoding can therefore be used as a first step in new species discovery and, as such, be used as a tool to help address the taxonomic impediment problem (e.g. Kekkonen and Hebert 2014).

Plecoptera is a neopteran exopterygote insect order characterised by a combination of mainly primitive characters, whose phylogenetic relationships with other insect orders are not completely resolved (Zwick 2000). Except in a few cases, they are amphibiotic animals, with eggs and nymphs occurring in freshwaters and adults inhabiting the terrestrial environment. The commonly called stoneflies are worldwide distributed, except in Antarctica and many islands and are usually associated with unpolluted and well-preserved waters, mainly rivers and streams, where they play important roles as part of their biota (Fochetti and Tierno de Figueroa 2008, Stewart 2009) contributing to important ecosystem services (DeWalt and Ower 2019). Their high vulnerability to environmental changes have driven stoneflies to be one of the most endangered groups of insects (Fochetti and Tierno de Figueroa 2008, Tierno de Figueroa et al. 2010).

A total of 3718 Plecoptera species have been described all over the world and 489 of them have been reported in Europe (DeWalt and Ower 2019). The European stonefly fauna, included in seven of the 16 existing families, is one of the best studied worldwide, but the degree of knowledge differs between countries. Of the Western European countries, Portugal is one of the less studied from a taxonomic and faunistic point of view. Furthermore, less than 50% of the Iberian Plecoptera have their DNA barcode sequenced. Although the first reports of stonefly species in Portugal date from the mid-XIXth century (Pictet 1841), only a few new records were added for this country during the following hundred years by authors such as Pictet A.E., Albarda, Kempny or Navás (in: Sánchez-Ortega et al. 2002). It was not until 1963 when the first exhaustive work on faunistic and chorology of stoneflies from Portugal, particularly for those of Serra da Estrela, was published as part of a wider study on the Iberian Peninsula (Aubert 1963). Afterwards, the main contributions to the knowledge of the taxonomy and/or faunistics of Plecoptera from Portugal were those of Zwick (1972), Whytton da Terra (1979), Berthélemy and Whytton da Terra (1980), Tierno de Figueroa et al. (1998), increasing the number of recorded species in the country from 25 to 53. More recently, the Portuguese fauna have been also studied in general publications for the Iberian Peninsula, such as those by Sánchez-Ortega et al. (2002) and Tierno de Figueroa et al. (2003), Tierno de Figueroa et al. (2015). According to Tierno de Figueroa et al. (2018), a total of 56 species were recorded in continental Portugal, without considering *Isoperla luzoni* Tierno de Figueroa & Vinçon, 2005, whose presence should be confirmed. No Plecoptera species has ever been collected in the Portuguese archipelagoes of Madeira and the Azores. Other areas from the Iberian Peninsula have been better studied regarding their Plecoptera fauna. Currently, 148 species of Plecoptera have been reported in the Iberian Peninsula and Balearic Islands (two of them endemic from the Balearic Islands), 144 species in Spain and 43 in Andorra (Tierno de Figueroa et al. 2018).

The InBIO Barcoding Initiative Database: contribution to the knowledge on DNA barcodes of Iberian Plecoptera dataset contains records of 71 specimens of Plecoptera collected in the Iberian Peninsula, all of which were morphologically identified to species level, for a total of 29 species. This is the first IBI dataset on freshwater insects available in the Global Biodiversity Information Facility (GBIF). All specimens have their DNA barcodes made publicly available in the Barcode of Life Data System (BOLD). Overall, this paper increases the available information on Iberian freshwater insects by sharing and publicly disseminating the distribution records and DNA barcodes of specimens from our reference collection.

## General description

### Purpose

This dataset aims to provide a first contribution to an authoritative DNA barcode sequences library for Iberian Plecoptera. Such a library should facilitate DNA-based identification of species for both traditional molecular studies and DNA-metabarcoding studies, as well as freshwater biomonitoring programmes and constitute a valuable resource for taxonomic research on Iberian Plecoptera and its distribution.

### Additional information

A total of 71 specimens of Plecoptera were collected and DNA barcoded (Suppl. materials 1, 2, 3). Sequences of cytochrome c oxidase I (COI) DNA barcodes are 658 bp long (Folmer region) with the exception of *Leuctra cazorlana*, from which a fragment of 325 bp was obtained. From the 29 species barcoded, 18 (62%) from seven families are new to the DNA barcode database BOLD at the moment of the release (marked with quotation mark ('') in the Species field of Table 1). Six additional BINs from these datasets are new to BOLD (marked with asterisk symbol (*) in BOLD BIN field of Table 1). Therefore, this dataset represents a significant contribution to enhance the species and genetic diversity of Iberian Plecoptera fauna represented in public libraries.

## Project description

### Title

The name “The InBIO Barcoding Initiative Database: contribution to the knowledge on DNA barcodes of Iberian Plecoptera” refers to the first data release of DNA barcodes and distribution data of stoneflies within the InBIO Barcoding Initiative.

### Personnel

Pedro Beja (project coordinator), Nuno Fonseca (project chair), Sónia Ferreira (taxonomist and IBI manager), Joana Paupério (IBI manager), Pedro Sousa (project technician), Filipa MS Martins (PhD student), Joana Veríssimo (PhD student), all affiliated to CIBIO-InBIO and Jose Manuel Tierno de Figueroa (taxonomist), Department of Zoology, University of Granada.

### Study area description

**Figure 1.**
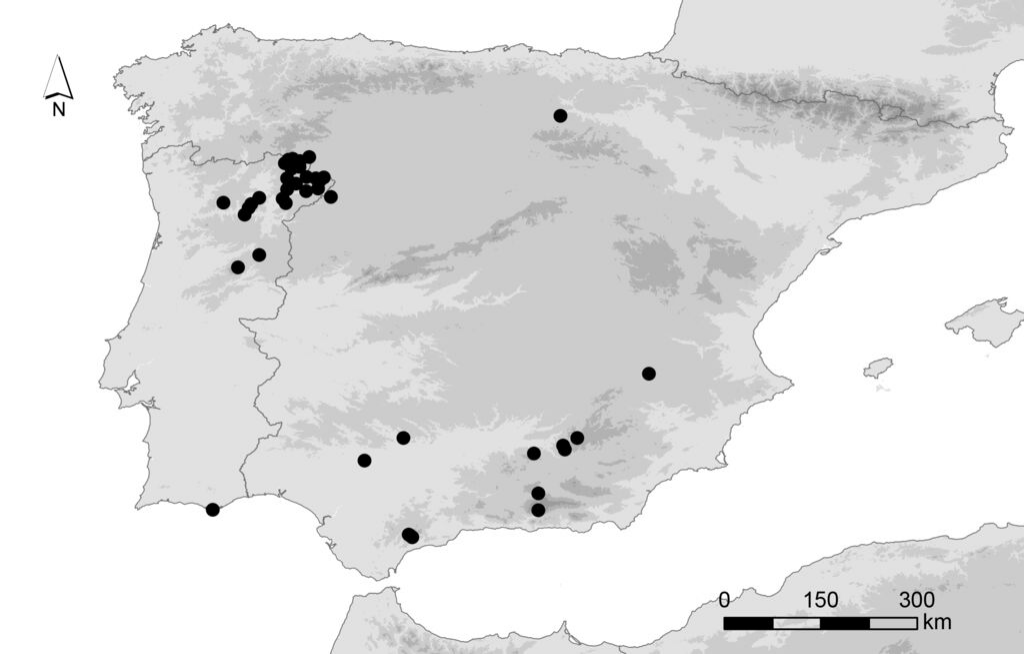
Map of the localities where Plecoptera samples were collected in the Iberian Peninsula.

Iberian Peninsula (Fig. 1)

### Design description

Plecoptera specimens were collected in the field, morphologically identified and DNA barcoded.

## Sampling methods

### Study extent

Iberian Peninsula.

### Sampling description

The studied material was collected in 40 different localities from the Iberian Peninsula (Suppl. materials 1, 2). Sampling was conducted between 2004 and 2018 on a wide range of habitats, using mainly hand-held sweep-nets or direct search for specimens. Collected specimens were examined in ethanol using a binocular stereoscopic microscope and they were stored in 96% ethanol for downstream molecular analysis. Morphological identification was performed using keys and descriptions from literature (mainly Tierno de Figueroa et al. 2003 and Tierno de Figueroa et al. 2015)

DNA extraction and sequencing followed the general pipeline used in the InBIO Barcoding Initiative (Ferreira et al. 2018). Briefly, genomic DNA was extracted from leg tissue using EasySpin Genomic DNA Tissue Kit (Citomed) following the manufacturer’s protocol. The cytochrome c oxidase I (COI) barcoding fragment (Folmer region) was amplified as two overlapping fragments (LC and BH), using two sets of primers: LCO1490 (Folmer et al. 1994) + Ill_C_R (Shokralla et al. 2015) and Ill_B_F (Shokralla et al. 2015) + HCO2198 (Folmer et al. 1994), respectively. The partial COI mitochondrial gene (Folmer region) was then sequenced in a MiSeq benchtop system. OBITools (https://git.metabarcoding.org/obitools/obitools) was used to process the initial sequences which were then assembled into a single 658 bp fragment using Geneious 9.1.8. (https://www.geneious.com).

### Quality control

All DNA barcodes sequences were compared against the BOLD database and the 99 top hits were inspected in order to detect possible issues due to contamination or misidentifications. Prior submission to GBIF, data were checked for errors and inconsistencies with OpenRefine 3.2 (http://openrefine.org).

### Step description

Specimens were collected in 40 different localities of the Iberian Peninsula. Sampling was conducted from 2004 to 2018 and consisted of direct search of specimens on rocks and vegetation of streams and river margins and in the use of entomological nets to intercept specimens in flight. Specimens collected were stored in 96% ethanol. A tissue sample (leg) was removed, from which DNA was extracted and the COI DNA barcode fragment was sequenced. Data generated were submitted to BOLD, GenBank and GBIF.

## Geographic coverage

### Description

Iberian Peninsula

### Coordinates

35.97 and 43.99 Latitude; 9.55 and 3.34 Longitude.

## Taxonomic coverage

### Description

This dataset is composed of data relating to 71 Plecoptera specimens. All specimens were determined to species level. Overall, 29 species are represented in the dataset. These species belong to 16 genera and seven families.

### Taxa included

**Table:** 

Rank	Scientific Name	Common Name
kingdom	Animalia	Animals
phylum	Arthropoda	Arthropods
class	Insecta	Insects
order	Plecoptera	Stoneflies
family	Capniidae	
family	Chloroperlidae	
family	Leuctridae	
family	Nemouridae	
family	Perlidae	
family	Perlodidae	
family	Taeniopterygidae	

## Temporal coverage

**Data range:** 2004-6-22 – 2018-5-19.

### Notes

The sampled material was collected in the period from 22 June 2004 to 19 May 2018

## Collection data

### Collection name

InBIO Barcoding Initiative

### Collection identifier

4ec2b246-f5fa-4b90-9a8d-ddafc2a3f970

### Specimen preservation method

“Alcohol”

### Curatorial unit

Voucher tube - 1 to 71, DNA extractions - 1 to 71

## Usage rights

### Use license

Creative Commons Public Domain Waiver (CC-Zero)

## Data resources

### Data package title

The InBIO Barcoding Initiative Database: contribution to the knowledge on DNA barcodes of Iberian Plecoptera

### Resource link

dx.doi.org/10.5883/DS-IBIPP01

### Number of data sets

1

### Data set 1.

#### Data set name

DS-IBIPP01 IBI-Plecoptera 01

#### Data format

dwc, xml, tsv, fasta

#### Number of columns

35

#### Download URL

http://www.boldsystems.org/index.php/Public_SearchTerms?query=DS-IBIPP01

#### Description

The InBIO Barcoding Initiative Database: contribution to the knowledge on DNA barcodes of Iberian Plecoptera dataset can be downloaded from the Public Data Portal of BOLD (http://www.boldsystems.org/index.php/Public_SearchTerms?query=DS-IBIPP01) in different formats (data as dwc, xml or tsv and sequences as fasta files). Alternatively, BOLD users can log-in and access the dataset via the Workbench platform of BOLD. All records are also searchable within BOLD, using the search function of the database.

The InBIO Barcoding Initiative will continue sequencing Iberian Plecoptera for the BOLD database, with the ultimate goal of comprehensive coverage. The version of the dataset, at the time of writing the manuscript, is included as Suppl. materials 1, 2, 3 in the form of one text file for record information as downloaded from BOLD, one text file with the collection and identification data in Darwin Core Standard format (downloaded from GBIF, Ferreira et al. 2020) and of a fasta file containing all sequences as downloaded from BOLD.

It should be noted that, as the BOLD database is not compliant with the Darwin Core Standard format, the Darwin Core formatted file (dwc) that can be downloaded from BOLD is not strictly Darwin Core formatted. For a proper Darwin Core formatted file, see http://ipt.gbif.pt/ipt/resource?r=plecoptera_01&amp;v=1.2 (Suppl. material 2).

All data are available in the BioStudies database (http://www.ebi.ac.uk/biostudies) under accession number S-BSST402.

**Data set 1.** 

Column label	Column description
processid	Unique identifier for the sample
sampleid	Identifier for the sample being sequenced, i.e. IBI catalogue number at Cibio-InBIO, Porto University. Often identical to the "Field ID" or "Museum ID"
recordID	Identifier for specimen assigned in the field
catalognum	Catalogue number
fieldnum	Field number
institution_storing	The full name of the institution that has physical possession of the voucher specimen
bin_uri	Barcode Index Number system identifier
phylum_taxID	Phylum taxonomic numeric code
phylum_name	Phylum name
class_taxID	Class taxonomic numeric code
class_name	Class name
order_taxID	Order taxonomic numeric code
order_name	Order name
family_taxID	Family taxonomic numeric code
family_name	Family name
subfamily_taxID	Subfamily taxonomic numeric code
subfamily_name	Subfamily name
genus_taxID	Genus taxonomic numeric code
genus_name	Genus name
species_taxID	Species taxonomic numeric code
species_name	Species name
identification_provided_by	Full name of primary individual who assigned the specimen to a taxonomic group
identification_method	The method used to identify the specimen
voucher_status	Status of the specimen in an accessioning process (BOLD controlled vocabulary)
tissue_type	A brief description of the type of tissue or material analysed
collectors	The full or abbreviated names of the individuals or team responsible for collecting the sample in the field
lifestage	The age class or life stage of the specimen at the time of sampling
sex	The sex of the specimen
lat	The geographical latitude (in decimal degrees) of the geographic centre of a location
lon	The geographical longitude (in decimal degrees) of the geographic centre of a location
elev	Elevation of sampling site (in metres above sea level)
country	The full, unabbreviated name of the country where the organism was collected
province_state	The full, unabbreviated name of the province ("Distrito" in Portugal) where the organism was collected
region	The full, unabbreviated name of the municipality ("Concelho" in Portugal) where the organism was collected
exactsite	Additional name/text description regarding the exact location of the collection site relative to a geographic relevant landmark

## Supplementary Material

BA2C0392-35B7-5E62-AE99-20D07837AA3E 10.3897/BDJ.8.e55137.suppl1 Supplementary material 1 IBI-Plecoptera 01 library - Specimen details Data type Record information - specimen data Brief description The file includes information about all records in BOLD for the IBI-Plecoptera 01 library. It contains collection and identification data. The data are as downloaded from BOLD, without further processing. File: oo_417920.txt https://binary.pensoft.net/file/417920 Sonia Ferreira, Jose Manuel Tierno de Figueroa, Lorenzo Quaglietta, José Manuel Grosso-Silva, Pedro B Lopes, Pedro Sousa, Pedro Beja

4807D8D9-D2DC-5F26-8E15-FE051964A7EB 10.3897/BDJ.8.e55137.suppl2 Supplementary material 2 IBI-Plecoptera 01 library - Specimen details - Darwin Core Standard Data type Record information - specimen data in Darwin Core Standard format Brief description The file includes information about all records in BOLD for the IBI-Plecoptera 01 library. It contains collection and identification data. The data are downloaded from GBIF, without further processing. File: oo_417919.txt https://binary.pensoft.net/file/417919 Sonia Ferreira, Jose Manuel Tierno de Figueroa, Filipa Martins, Joana Veríssimo, Pedro Sousa, Pedro Beja

2A18AAB5-E066-5134-BEB0-88F1A3729241 10.3897/BDJ.8.e55137.suppl3 Supplementary material 3 IBI-Plecoptera 01 library - DNA sequences Data type Genomic data, DNA sequences Brief description COI sequences in fasta format. Each sequence is identified by the BOLD ProcessID, species name, marker and GenBank accession number, separated by pipe. The data are as downloaded from BOLD. File: oo_417901.fas https://binary.pensoft.net/file/417901 Sonia Ferreira, Jose Manuel Tierno de Figueroa, Filipa Martins, Joana Veríssimo, Joana Paupério, Pedro Sousa, Pedro Beja

## Figures and Tables

**Table 1.** List of species that were collected and DNA barcoded within this project. " Indicate species new to BOLD database and * new BINs in BOLD database.

Family	Species	IBI code	BOLD code	BOLD BIN	GenBank	BOLD BIN
Capniidae	*Capnioneura libera*"	INV00770	IBIPP042-20	AEC8556	MT407216	AEC8556
	*Capnioneura mitis*"	INV02034	IBIPP045-20	AEC7867	MT407200	AEC7867
		INV02035	IBIPP046-20		MT407211	AEC7867
		INV02036	IBIPP047-20		MT407226	AEC7867
	*Capnioneura petitpierreae*"	INV03768	IBIPP079-20	AEC8557	MT407228	AEC8557
	*Capnopsis schilleri*	INV03770	IBIPP009-19	ADV3255*	MT407199	ADV3255
Chloroperlidae	*Chloroperla acuta*"	INV06355	IBIPP099-20	AEC9580	MT407268	AEC9580
	*Siphonoperla torrentium*	INV00467	IBIPP002-19	ADT9540	MT407262	ADT9540
		INV03933	IBIPP011-19		MT407247	ADT9540
		INV03934	IBIPP012-19		MT407207	ADT9540
		INV06330	IBIPP095-20		MT407206	ADT9540
		INV06354	IBIPP098-20		MT407244	ADT9540
		INV06359	IBIPP100-20		MT407208	ADT9540
		INV06360	IBIPP101-20		MT407265	ADT9540
Leuctridae	*Leuctra andalusiaca*"	INV03771	IBIPP010-19	ACX4018	MT407238	ACX4018
	*Leuctra cazorlana*"	INV03775	IBIPP082-20		MT407235	
	*Leuctra franzi*"	INV03718	IBIPP076-20	AEC8030	MT407220	AEC8030
	*Leuctra geniculata*	INV02033	IBIPP007-19	AAM4209	MT407255	AAM4209
		INV03720	IBIPP008-19		MT407267	AAM4209
	*Leuctra iliberis*"	INV02025	IBIPP052-20	AEC6493	MT407209	AEC6493
		INV02026	IBIPP044-20		MT407261	AEC6493
	*Leuctra major*	INV03719	IBIPP077-20	ACB2856	MT407242	ACB2856
	*Tyrrhenoleuctra lusohispanica*"	INV00350	IBIPP017-19	ACD6989	MT407256	ACD6989
		INV00405	IBIPP023-19		MT407263	ACD6989
		INV02875	IBIPP048-20		MT407257	ACD6989
		INV02926	IBIPP074-20		MT407222	ACD6989
Nemouridae	*Amphinemura guadarramensis*"	INV06379	IBIPP102-20	AEC7918	MT407223	AEC7918
	*Amphinemura sulcicollis*	INV00773	IBIPP004-19	AAM5074	MT407202	AAM5074
	*Nemoura cinerea*	INV00367	IBIPP033-20	ADS8217*	MT407251	ADS8217
		INV00368	IBIPP001-19		MT407266	ADS8217
	*Nemoura lacustris*	INV00389	IBIPP020-19	AEB8934*	MT407258	AEB8934
		INV00392	IBIPP021-19		MT407215	AEB8934
		INV00345	IBIPP016-19	AEB9369*	MT407225	AEB9369
		INV00382	IBIPP018-19		MT407224	AEB9369
		INV00385	IBIPP019-19		MT407214	AEB9369
		INV00454	IBIPP024-19		MT407212	AEB9369
		INV04847	IBIPP037-20		MT407250	AEB9369
		INV06416	IBIPP103-20		MT407264	AEB9369
		INV00404	IBIPP022-19	AEC1305*	MT407237	AEC1305
	*Protonemura alcazaba*	INV02028	IBIPP067-20	AEC7157*	MT407227	AEC7157
	*Protonemura meyeri*	INV02031	IBIPP005-19	ADS3277	MT407253	ADS3277
		INV02032	IBIPP006-19		MT407252	ADS3277
Perlidae	*Eoperla ochracea*"	INV02021	IBIPP043-20	AEC9593	MT407249	AEC9593
	*Perla madritensis*"	INV00640	IBIPP026-19	AEB9929	MT407260	AEB9929
		INV00677	IBIPP027-19		MT407245	AEB9929
		INV00688	IBIPP028-19		MT407205	AEB9929
		INV00764	IBIPP039-20		MT407233	AEB9929
		INV05226	IBIPP038-20		MT407232	AEB9929
	*Perla marginata*	INV04280	IBIPP013-19	AAL2357	MT407210	AAL2357
		INV04281	IBIPP014-19		MT407221	AAL2357
Perlodidae	*Guadalgenus franzi*"	INV00344	IBIPP015-19	AEB8450	MT407248	AEB8450
		INV00355	IBIPP055-20		MT407230	AEB8450
		INV00358	IBIPP032-20		MT407269	AEB8450
	*Hemimelaena flaviventris*"	INV00507	IBIPP025-19	AEC1314	MT407236	AEC1314
		INV00766	IBIPP040-20		MT407243	AEC1314
		INV02925	IBIPP031-19		MT407218	AEC1314
	*Isoperla bipartita*"	INV00828	IBIPP029-19	AEC0487	MT407203	AEC0487
		INV00850	IBIPP030-19		MT407231	AEC0487
	*Isoperla grammatica*	INV00183	IBIPP053-20	AEC9627	MT407254	AEC9627
		INV00479	IBIPP034-20		MT407240	AEC9627
		INV00500	IBIPP003-19		MT407213	AEC9627
		INV00548	IBIPP035-20		MT407234	AEC9627
		INV00767	IBIPP058-20		MT407204	AEC9627
		INV00768	IBIPP059-20		MT407219	AEC9627
		INV03931	IBIPP084-20		MT407201	AEC9627
		INV03932	IBIPP085-20		MT407246	AEC9627
		INV04831	IBIPP036-20		MT407217	AEC9627
		INV07241	IBIPP104-20		MT407229	AEC9627
	*Isoperla pallida*"	INV03772	IBIPP050-20	AED0411	MT407259	AED0411
Taeniopterygidae	*Brachyptera auberti*"	INV04837	IBIPP092-20	AEC8527	MT407241	AEC8527
	*Rhabdiopteryx thienemanni*"	INV03773	IBIPP051-20	AEC7722	MT407239	AEC7722
